# Hong Kong’s role in global health: Public opinion of official development assistance

**DOI:** 10.1371/journal.pone.0207687

**Published:** 2018-12-04

**Authors:** Chi Him Lee, Brian Tse, Nathaniel Lai, William Goggins, Larry Baum, E Anthony S. Nelson

**Affiliations:** 1 Graduate Institute of International and Development Studies, Geneva, Switzerland; 2 Independent researcher, Hong Kong, Hong Kong Special Administrative Region, People’s Republic of China; 3 Jockey Club School of Public Health and Primary Care, The Chinese University of Hong Kong, Hong Kong Special Administrative Region, People’s Republic of China; 4 Centre for Genomic Sciences, The University of Hong Kong, Hong Kong Special Administrative Region, People’s Republic of China; 5 Department of Paediatrics, The Chinese University of Hong Kong, Hong Kong Special Administrative Region, People’s Republic of China; University of Cambridge, UNITED KINGDOM

## Abstract

Governments in high income countries allocate funding for Official Development Assistance (ODA), and population-based surveys tend to show support for the concept of affluent nations assisting the development of poorer regions. A public opinion survey was conducted in Hong Kong to: (1) assess public support for foreign aid for social development and Hong Kong's current Disaster Relief Fund (DRF); and (2) assess how much respondents thought should be contributed to foreign aid for social development and/or DRF. Interviewers conducted a random telephone survey of Cantonese-speaking Hong Kong citizens aged 18 or above during 2017. Of the 1004 individuals surveyed, 55% (552) agreed that a portion of the government budget should be allocated to the DRF and 37% (372) disagreed. The mean and the median amount of the government budget suggested to be allocated were 5.1% and 2.4% respectively. However only 16% (164) supported the government giving foreign aid for social development, with 79% (793) not supporting, and 5% (47) undecided. The suggested portions of government budget that should be allocated for this purpose were 1.5% (mean) and 0.0% (median). The degree of support for DRF and foreign aid for social development was associated with both age (DRF P < 0.0005; foreign aid for social development P < 0.0005) and education (DRF P = 0.010; foreign aid for social development: P < 0.0005). There was little support for foreign aid for social development amongst the Hong Kong public, in contrast to similar surveys in other countries, but this could be related to the lack of a local tradition of providing ODA to foreign countries. Most respondents supported the current DRF and would like to see a greater proportion of government budget allocated.

## Introduction

The Development Aid Committee (DAC) of the Organization of Economic Cooperation and Development (OECD) defines Official Development Assistance (ODA) as *“[f]lows of official financing administered with the promotion of the economic development and welfare of developing countries as the main objective*, *and which are concessional in character with a grant element of at least 25 percent (using a fixed 10 percent rate of discount)*”. ODA may be bilateral (between donor and receipt countries) or multilateral when it is channelled through international organisations such as the United Nations and its organisations, the International Committee of the Red Cross, and the Global Fund to Fight AIDS, Tuberculosis and Malaria, etc. According to OECD, the total amount of ODA from DAC member countries has increased from $36 billion in 1960 to $144 billion in 2017 [1], and non-DAC countries, such as Qatar and China, have emerged as providers of development finance [2]. In 1970 the United Nations General Assembly passed Resolution 2626, that included the goal that each “economically advanced country will progressively increase its official development assistance to the developing countries and will exert its best efforts to reach a minimum net amount of 0.7 per cent of its gross national product at market prices by the middle of the Decade.” The 2015 UN Sustainable Development Goals reaffirmed the call for developed economies to contribute at least 0.7% of Gross National Income (GNI) as ODA (Goal 17.2).

The purpose of development aid is debated, with some arguing it often strengthens the diplomatic standing of the donor country and promotes capitalistic values rather than supporting social and economic development [3] (page 3). The purpose of foreign aid can be attributed to domestic politics, which can be a key factor influencing public opinion. Lancaster suggests, “the widely shared values and worldviews in donor countries, especially about the appropriate role of the state in society and the role of the donor country in the world, affect public attitude toward the role of the donor country in the world, affect public attitudes towards the legitimacy and use of aid and, more indirectly, toward the interests competing for control over aid” [3]. An empirical study showed that Nordic countries are among the most altruistic aid providers, whereas the development aid from other countries such as Australia, Italy and France tends to have strings attached [4]. Public opinion can influence the foreign policy agenda and parameters [5], the volume of aid offered [6], the channels through which donor countries provide foreign aid [7], and the selection of recipient countries [8]. Multiple drivers can explain the variation of attitudes towards foreign aid across countries, such as religiosity, international affairs savviness, trust towards peers and institutions, racial paternalism, economic ideology (such as that towards the cause of poverty), individual material factors (such as wealth, education and professions), and perceived aid effectiveness [9,10].

Despite their limitations, surveys conducted to examine public attitude of donor countries towards aid have mainly shown that there is a consensus among the public of major donor countries that affluent countries should assist the development of poorer regions. A majority of people in almost all of the 47 countries in a 2007 survey felt that “the wealthier nations of the world are not doing enough to help the poorer nations of the world with such problems as economic development, reducing poverty, and improving health”: 63% in Japan, 69% in the United States, 75% in Germany, and 81% in France [11]. In a 2016 survey, 66% of Japanese favoured increasing foreign aid to developing countries [12]. When asked whether their country is spending “too much”, “too little” or “the right amount” on “economic aid to other countries”, a 2003 survey in Europe and the United States on “economic aid to other countries” found that 37% of respondents felt that their country was spending the right amount, 19% felt that their country was spending too much, and 31% felt that the expenditure was too little [11]. Further, respondents tend to overestimate the amount of aid their countries offered to foreign countries, even when the budgets of other government programs are offered as comparisons [9,13]. But in a 2005 survey of 10 mostly European countries, when people were informed of the amount of their tax money going to foreign aid, 46% believed their country’s foreign aid level was about right. Most of the remaining respondents believed that the spending was too low (35%), with a significantly smaller number of respondents believing that their government was spending too much (9%) [11].

China has provided US$39 billion of ODA as grants and loans since the 1950s and about US$200–700 million annually between 2000 and 2006, becoming a net donor around 2005 [14,15]. Between 2000 and 2014, China gave US$350 billion in aid to 140 countries and territories, and it has become the largest official donor in some countries [16]. China has increasingly assumed a role in development finance, although not within the traditional DAC definition of ODA, with less than 25% of the financial assistance being concessional, and the remainder being largely commercial loans, often for energy and infrastructure projects. Nevertheless, China’s ODA allocations are not necessarily more self-interested than the West [17], and its projects have fostered economic development, with a doubling of ODA from China raising economic growth in recipient countries by an estimated average of 0.94 percentage points, compared to 1.4 percentage points for ODA from OECD-DAC countries [16]. Hong Kong is a Special Administrative Region of China with its own financial system and government budget. Government taxation and spending in Hong Kong is separate from that in the rest of China, therefore ODA from the rest of China and Hong Kong are separate, and taxes raised in Hong Kong do not support ODA from the government of China. In 2017 Hong Kong's GDP per capita was US$45,900 (HK$360,000), ranking it as one of the most affluent populations globally [18]. Under the "One country, Two systems" principle, Hong Kong's government is not responsible for foreign affairs [19]. As such Hong Kong has no formal ODA programme, but Hong Kong's government does contribute a variable amount of short-term aid through its Disaster Relief Fund (DRF). Managed directly by the office of the Chief Secretary of Hong Kong, the DRF “*provides a ready mechanism for Hong Kong to respond swiftly to international appeals for humanitarian aid in relief of disasters that occur outside Hong Kong*”. The DRF receives applications from relief organisations and evaluates needs accordingly [20]. According to the DRF’s guidelines, grants are only made in cases of specific disasters–such as earthquakes or floods–and not on-going problems, like malaria [21]. The total amount of grants in recent years have ranged from a minimum of HK$41.5 million (in 2011–2) to a maximum of HK$354 million (in 2010–1) [22][23]. This equates to 0.011% to 0.12% of Hong Kong's government budget (HK$371 billion in 2011–2 and HK$304 billion in 2010–1, respectively), or 0.0021% to 0.020% of GNI (HK$1.99 trillion in 2011 and HK$1.81 trillion in 2010, respectively). For comparison, Hong Kong's government allocates 18.1%, 17.8%, and 14.3% of its budget (or 3.5%, 3.4% and 2.7% of Hong Kong’s GNI) to infrastructure, education, and health, respectively [24].

If Hong Kong were to give 0.7% of GDP as ODA, the amount would be HK$18.6 billion (US$2.4 billion) per year, a substantial increase (1.7%) of the total ODA from DAC countries (2017 data), and similar to the contribution of either Australia, South Korea, or Switzerland [25]. Despite this potential contribution, Hong Kong does not currently give any ODA and it is possible that a lack of information on ODA locally could negatively impact on public perceptions and support. The existing literature suggests that people tend to view aid more favourably after learning basic facts regarding aid. Hong Kong's unique situation provides the opportunity to assess the level of public support for ODA in a population where there is no tradition of giving ODA. We therefore conducted a public opinion survey to (1) assess the level of public support for the notion of ODA, or the expansion of the current disaster relief mechanism; and (2) assess how much respondents thought should be contributed to ODA or increased DRF.

## Materials and methods

Ethics approval was granted by the Survey and Behavioural Research Ethics Committee of the Chinese University of Hong Kong. Participants did not provide written consent since this was a telephone public opinion survey and obtaining written consent would not be feasible. Verbal consent to participate in the survey was asked at the time of the telephone contact. Those participants who refused to participate in the survey did not give their verbal consent to participate. The ethics committee was aware that this was a public opinion survey and that verbal consent would be obtained at the time of the telephone contact.

This public opinion survey was conducted in collaboration with the Public Opinion Programme of The University of Hong Kong. Interviewers conducted a random telephone survey of 1004 Cantonese-speaking Hong Kong citizens aged 18 or above. 95.8% of the Hong Kong population speaks Cantonese as the usual language or as another language or dialect [26]. The phone calls were made from 6 pm to 10 pm (80% from 7 pm to 9 pm) from Monday to Thursday, 19–22 June 2017. Weighting was applied to adjust the distribution of respondents to match that of the Hong Kong population of age 18 or above with respect to gender, age, and education. The sampled response rate was 71.0% (standard error: <1.6% at a 95% confidence level).

Opinions on Hong Kong’s DRF and potential ODA, as well as how much of the government budget should be allocated to these, were surveyed with two structured questions (S1 Appendix). To offer realistic comparisons, the portions of government expenditure on education, health and infrastructure were stated. Introduction to the concepts of ODA and the DRF were provided in the preamble to the questions (S1 Appendix).

To check the representativeness of our sample we compared the distributions of age, gender, and education levels of our survey respondents with data from the Hong Kong Census and Statistics Department from the 2016 by-census using the chi-square goodness-of-fit test. All results were then weighted based on the distribution of demographics.

Regression was used to analyze the effect of demographic factors on the survey results. For each of the two outcomes, two models were fitted: (1) a logistic regression model for agree vs. disagree for whether or not any aid should be given, and (2) a linear regression for the recommended percentage among those who agreed to give aid. SPSS (IBM Corp.) and PSPP (http://www.gnu.org/software/pspp/) were used for statistical analysis.

## Results

**Fig 1:**
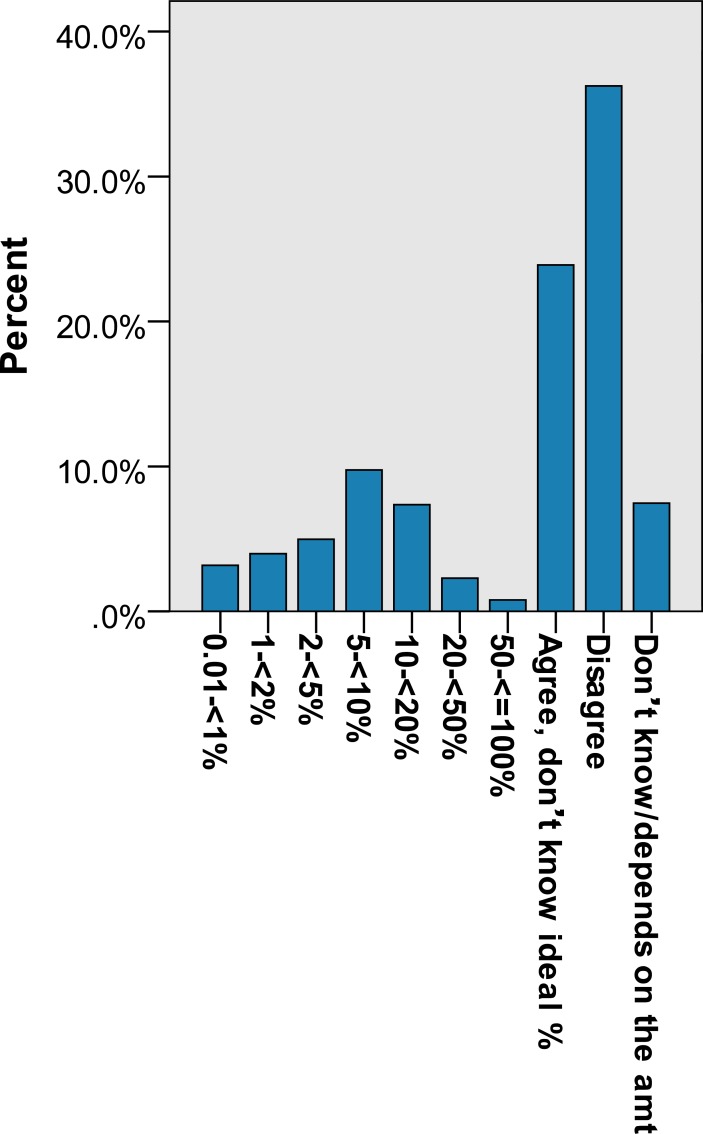
**Percent of n = 925 respondents' answers to:** Do you agree or disagree that some government budget should be allocated to the Disaster Relief Fund? If yes, what would be the ideal percentage?

**Fig 2:**
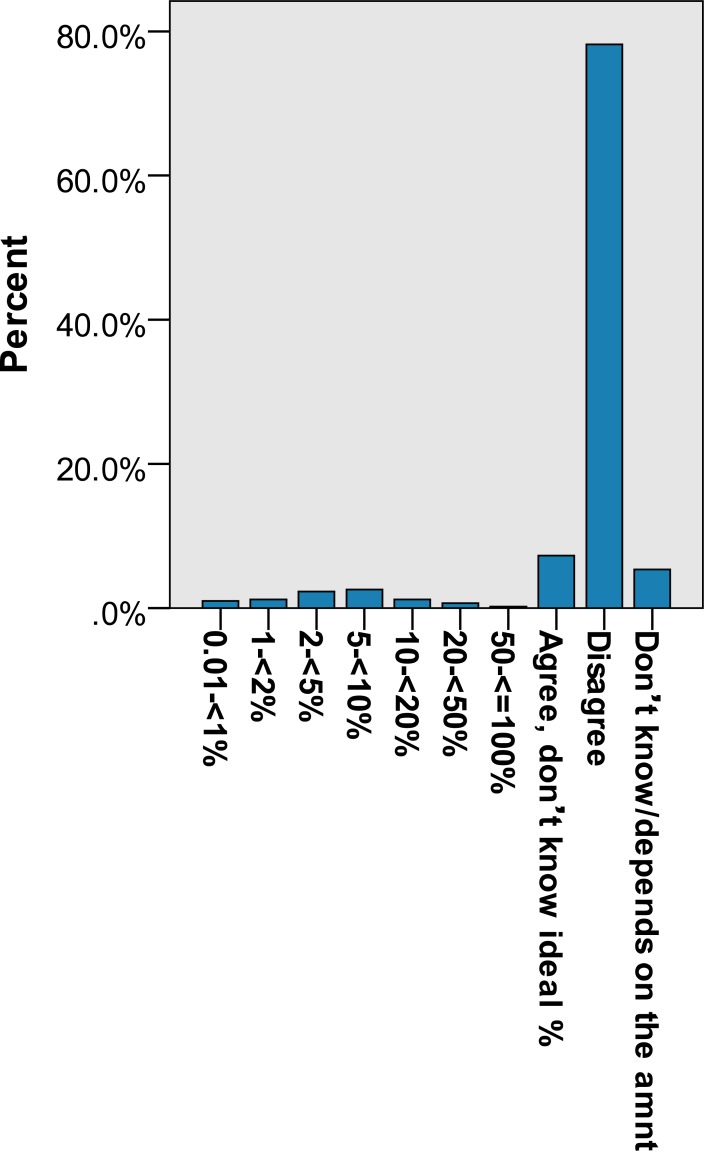
**Percent of n = 950 respondents' answers to:** Do you agree or disagree that some government budget should be allocated to Foreign Aid for Social Development? If yes, what would be the ideal percentage?

Of the 1004 individuals surveyed, 552 (55.0%) agreed that a portion of the government budget should be allocated to the DRF while 372 (37.0%) disagreed and 8% (80) answered ‘don’t know/not sure/depends on amount’. When asked about the amount of the government budget that should be allocated, the mean and the median were 5.1% and 2.4% respectively. For ODA, 164 (16.4%) agreed that there should be foreign aid for social development, 793 (79.0%) disagreed and 5% (47) answered ‘don’t know/not sure/depends on amount’. The mean portion of allocated government budget suggested for foreign aid for social development was 1.5% and the median was 0.0%. Detailed responses to the questions on attitudes towards DRF and ODA are shown in Figs 1 and 2, with the possible answers including agree with aid but don’t know the percentage, disagree with aid, don’t know, and for those who specified the percentage, the actual percentage they thought should go to aid (grouped).

All the results listed were weighted based on the distribution of demographics. We found that, compared to the general population, our survey respondents were substantially and significantly more likely to be in the older age groups, slightly, but significantly, more likely to be female, and moderately and significantly more likely to have a tertiary education. There were 437 males and 567 females surveyed. To assess the associations of the results with occupation, respondents were grouped into six categories: professionals and semi-professionals (209), clerks and service workers (177), production workers (49), students (49), housewives (149) and others (352). Six age groups were analysed: 18–29 years (127), 30–39 (89), 40–49 (150), 50–59 (199), 60–69 (223), and 70 or above (208). Nine educational levels were considered: primary school or below (189), junior secondary (147), senior secondary (237), diploma (53), matriculation (25), some tertiary (75), bachelor’s degree (210), master’s degree (55), and doctoral degree (9).

The results of univariable analyses examining associations between agree vs. disagree for government support for a DRF are shown in Table 1. Age was very significantly associated with this variable, with highest support being seen among those 40–49 and 70+. Education was not significantly associated, although support was higher among those with postgraduate education and lowest among those with tertiary, matriculation, or senior secondary education. Occupation was not significantly associated with support. These results were confirmed with logistic regression (Table 1); only age group was significant, and adjusted odds ratios indicated the strongest support was among those aged 40–49 and 70+ (adjusted odds ratios around 2.5). In linear regression (Table 2) again only age was significant, but in this case the older groups all preferred less than the younger group, and those 40–49 the least of all. Education was not significant (P = .15), but those with tertiary education did prefer less than those with senior secondary education. There was not much difference between the other groups.

**Table 1:** Univariable and multiple logistic regression analysis of agree vs. disagree with government financial support for a disaster relief fund.

	Agree	Disagree	P (agree vs. disagree)	Adjusted Odds Ratio (95% CI)	P-value
Covariate					
**Age**			< .0001		< .0001
20–29	64 (52%)	58 (48%)		1 (ref)	
30–39	44 (55%)	36 (45%)		1.27 (0.66, 2.42)	
40–49	95 (69%)	42 (31%)		2.43 (1.33, 4.45)	
50–59	108 (59%)	74 (41%)		1.55 (0.86, 2.78)	
60–69	107 (52%)	98 (48%)		0.98 (0.52, 1.86)	
70+	134 (74%)	48 (26%)		2.49 (1.21, 5.09)	
**Education**					.44
Primary or below	96 (63%)	57 (37%)		1.02 (0.63, 1.65)	
Junior Secondary	91 (66%)	47 (34%)		1.31 (0.83, 2.08)	
Senior Secondary	130 (59%)	90 (41%)		1 (ref)	
Matriculation	45 (59%)	31 (41%)		1.14 (0.65, 1.99)	
Tertiary	148 (57%)	113 (43%)		1.01 (0.67, 1.52)	
Postgraduate	42 (70%)	18 (30%)		1.80 (0.92, 3.49)	
					.46
**Occupation**					
Clerk/Service	93	76		0.90 (0.57, 1.41)	
Housewife	79	54		0.98 (0.57, 1.68)	
Other	201	108		1.30 (0.77, 2.19)	
Production	30	18		1.22 (0.59, 2.50)	
Professional	120	81		1 (ref)	
Student	29	19		1.70 (0.78, 3.70)	
**Total**	552	356		1.01 (0.75, 1.38)	

**Table 2:** Linear regression for amount of aid supported for a disaster relief fund among those who support this fund.

	Beta (95% CI)	P-value
Covariate		
Age		.012
20–29	0 (ref)	
30–39	-3.57 (-7.20, 0.05)	
40–49	-5.47 (-8.64, -2.28)	
50–59	-4.61 (-7.83, -1.40)	
60–69	-4.39 (-7.84, -0.94)	
70+	-2.61 (-6.26, 1.04)	
**Education**		.15
Primary or below	-0.05 (-2.39, 2.29)	
Junior Secondary	0.97 (-1.21, 3.14)	
Senior Secondary	0 (ref)	
Matriculation	-0.70 (-3.60, 2.20)	
Tertiary	-2.28 (-4.37, -0.19)	
Postgraduate	-0.80, (-3.78, 2.18)	
**Occupation**		.22
Clerk/Service	0.99 (-1.33, -3.30)	
Housewife	0.24 (-2.44, 2.93)	
Other	-1.38 (-3.89, 1.13)	
Production	-1.80 (-5.26, 1.66)	
Professional	0 (ref)	
Student	-3.01 (-7.18, 1.16)	

For agree vs disagree with government support for foreign aid for social development (Table 3), age was not significantly associated with this outcome in univariable analysis (P = .41), but the youngest and oldest groups were generally more supportive. Education was also not significant (P = .19). Support was highest among those with a postgraduate education (24%) and those with a primary education (21%) and lowest for those with senior secondary education (12%). Occupation was also not significant (P = .66), although students (23%) and housewives (21%) were somewhat more supportive. These results were also supported by the logistic regression results (Table 3). Although none of the predictors were significant, all age groups except 70+ were less likely to support foreign aid for social development than the youngest group, and those with postgraduate education were more likely to support foreign aid for social development (adjusted odds ratio = 2.18; 95% confidence interval = 0.997, 4.75). In the linear regression analysis on amount of foreign aid for social development (Table 4), while the overall age association was not significant (P = .14), all of the age groups except the 30–39 year old group supported significantly less foreign aid for social development than the youngest group. Education was significant (P = .018), with the more educated groups supporting less foreign aid for social development than those with senior secondary education. Occupation was not significant.

**Table 3:** Univariable and multiple logistic regression analysis of agree vs. disagree with government financial support for foreign aid for social development.

	Agree	Disagree	P (agree vs. disagree)	Adjusted Odds Ratio (95% CI)	P-value
Covariate					
**Age**			.41		.60
20–29	25	97		1 (ref)	
30–39	14	68		0.86 (0.37, 1.97)	
40–49	24	120		0.85 (0.40, 1.81)	
50–59	27	159		0.74 (0.34, 1.58)	
60–69	32	179		0.80 (0.35, 1.81)	
70+	39	140		1.25 (0.51, 3.05)	
**Education**			.19		.37
Primary or below	35	128		1.69 (0.92, 3.11)	
Junior Secondary	25	116		1.52 (0.83, 2.79)	
Senior Secondary	27	192		1 (ref)	
Matriculation	14	62		1.47 (0.70, 3.07)	
Tertiary	46	220		1.37 (0.78, 2.41)	
Postgraduate	14	45		2.18 (1.00, 4.75)	
**Occupation**			.66		.74
Clerk	25	147		0.94 (0.52, 1.71)	
Housewife	29	112		1.30 (0.66, 2.55)	
Other	53	264		0.84 (0.43, 1.63)	
Production	8	39		1.11 (0.44, 2.83)	
Professional	35	165		1 (ref)	
Student	11	36		1.31 (0.51, 3.38)	
**Total**	161	763			

**Table 4:** Linear regression analysis for amount of aid supported for foreign aid for social development among those who support aid.

	Beta (95% CI)	P-value
Covariate		
Age		.14
20–29	0 (ref)	
30–39	-4.68 (-12.56, 3.20)	
40–49	-8.30 (-15.20, -1.41)	
50–59	-2.90 (-14.80, -0.98)	
60–69	-9.08 (-16.09, -2.06)	
70+	-8.21 (-15.90, -0.51)	
Education		.018
Primary or below	-1.66 (-7.23, 3.91)	
Junior Secondary	3.82 (-1.92, 9.57)	
Senior Secondary	0 (ref)	
Matriculation	-5.25 (-11.96, 1.47)	
Tertiary	-5.75 (-10.97, -0.52)	
Postgraduate	-5.48 (-12.57, 1.60)	
**Occupation**		.28
Clerk	1.87 (-3.59, 7.33)	
Housewife	-0.72 (-7.02, 5.58)	
Other	1.80 (-3.89, 7.48)	
Production	-3.20 (-11.56, 5.16)	
Professional	0 (ref)	
Student	-6.89 (-15.28, 1.49)	

## Discussion

There was a generally favourable view of the Disaster Relief Fund. This is consistent with previous findings in other countries showing that support for foreign aid is based on the assumption that the aid would be spent on remedying humanitarian crises [27], and it is relevant to note that Hong Kong’s DRF approves grants only for specific disasters but not ongoing humanitarian problems [20]. The preferred percentage is far higher than the existing amount, which stands at less than 0.1% and often less than 0.01% of Hong Kong's GNI. This result is consistent with the often vast overestimation of actual aid amounts in surveys of other developed economies [9,13,28]. The strong public support for the DRF might be attributed to the vivid images one observes in times of natural disasters and to the longstanding tradition of donating for relief of disaster victims.

However, most surveyed citizens rejected Hong Kong having a role in financial support for social programmes as part of ODA, with less than one-fifth of the respondents approving the notion. Considering the charity given by Hong Kong people–ranked 21 out of 139 countries in proportion of people who donate to charity, with Hong Kong taxpayers reporting HK$7 billion of donations to charities in 2015 –this is a surprising finding [29,30]. Yet it is consistent with the theory that a lack of information about aid reduces public support for aid. Hong Kong lacks a local tradition of ODA, with Hong Kong having had no independent foreign policy both during British colonial times and since 1997 as part of China. Thus the concept of ODA could be unfamiliar to the local population, perhaps leading people in Hong Kong to choose personal donation as a way of supporting humanitarian causes. Multiple reports express the idea that public knowledge regarding aid raises support for aid, and surveys confirm this [31]. A report on the UK Public Opinion Monitor listed as a key finding that “Informing people about poverty impacts on their support for aid in specific situations” and found that the low level of support for aid to India doubled from 15% to 30% after informing respondents of the degree of poverty in India [32]. In South Korea, people who knew about the Millennium Development Goals were more likely to favour foreign aid [33]. When US respondents, who thought—on average—that foreign aid was 18% of the government’s spending, were informed that aid was actually <1%, their support for aid rose from 51% to 79%; the author says, “the point here is that, with complex issues such as foreign aid, there are great variances in the results of public opinion polls depending on levels of awareness [34].”

Hong Kong’s ambiguous standing in the international community may also affect its residents’ opinions towards ODA, as other surveys have found that populations show a higher level of support for development aid when the survey question is framed as part of the political entity taking greater responsibility for dealing with international threats. Another reason for the low level of support, as observed in previous surveys worldwide, is that people tend to put a higher priority on solving local issues than helping the wider global community [27]. Finally, the portrayals of refugees and asylum seekers as criminals in local media may have negatively impacted on support for humanitarian aid [35].

Worldwide, residents of rich countries tend to approve of ODA. In a 2001 survey of 13 OECD DAC countries, 80% of people supported the principle of giving aid to developing countries [27]. Support for a particular level of aid, however, may vary widely among countries. One factor in this variation might be inertia: a tendency to support the status quo. People may favour the current level of aid, regardless of whether it is high or low. Consistent with this possibility is the finding of a survey asking people in 10 countries if they felt that their country’s current level of foreign aid was about right, too high, or too low. The plurality in most countries answered “about right” despite the fact that aid per person ranged over more than an order of magnitude among these countries: from about US$9 in Spain to US$93 in Sweden (the exceptions were Spain and Andorra, where the plurality felt aid was too low, Sweden, where equal numbers felt aid was too low or about right, and Japan, where the plurality didn’t know or didn’t answer) [11]. Many Hong Kong residents may likewise support the status quo: zero ODA.

Consistent with the theory that absence of knowledge about ODA may reduce support for it, 67% of surveyed people in the US felt the US spends too much on foreign aid, but that proportion plunged to 28% after people were informed of several facts regarding aid (the facts were that the average American believes the foreign aid budget is 25 percent of the total federal government budget; the U.S. foreign aid budget is less than 1 percent of the total budget; only 1 percent of the U.S. foreign aid budget goes to operating costs of U.S. government agencies; the United States provides $30 billion for programs that assist the needy around the world, while around $663 billion goes to military spending; and in 1970, the world’s rich countries agreed to give 0.7 percent of their gross national income as foreign aid, and although most countries have not reached this goal, five have exceeded it, though, at 0.2 percent, the United States is far behind [36]. To test whether the low support for ODA by Hong Kong residents might reflect a similar absence of knowledge, a corresponding survey could be performed in Hong Kong, with the expectation that more respondents would favour ODA, or higher levels of ODA, after learning such information.

The differences between demographic groups showed interesting findings. Younger respondents tend to be more supportive of DRF and ODA. One explanation for this may be a higher level of social awareness among the younger generation. Interestingly, those with low and high levels of education preferred the highest levels of DRF and ODA while those with middle levels of education preferred less aid.

There are some limitations to the study. Additional data may assist in interpreting the results of the survey. Qualitative techniques such as interviews and focus groups would provide more detailed insight into the factors determining the level of support of people for DRF or ODA. We have reduced the effects of some well-documented surveying problems by introducing the concepts of DRF and ODA and presenting DRF expenditure in relation to the expenditure in other sectors, but other issues, such as courtesy bias, are hard to avoid. We kept the interview brief to avoid boring respondents and perhaps losing their attention or having them end the call. However, such short explanations of the DRF and social development aid may not adequately convey the meaning of these types of aid, and demographic moderators other than age, gender, education, and occupation, such as political views, might have carried useful information. Lack of familiarity with ODA might have reduced support for it in Hong Kong, but our survey did not test for this possibility. Future surveys may include countries that give ODA, allowing comparison with Hong Kong regarding the level of support for ODA. The order of the questions may affect the view on DRF relative to ODA. Respondents were first asked about DRF and then about ODA. Perhaps after expressing support for one program, respondents were less inclined to allocate additional money to support another, related program. Future surveys may shuffle the questions to enable comparison of responses between the subsets of respondents given different orders of questions. The level of support for aid may partly reflect public opinion of government. Future surveys could attempt to control for this by asking about trust in government and partisan ideology. Previous surveys have also found that the wordings of the questions–such as whether the purpose of the aid is specified–may significantly influence the responses [11]. Furthermore, while some previous surveys in other countries had told respondents how much of their tax money actually goes to foreign aid, this survey chose not to do so as the survey also aims to determine the amount of the government budget which people are willing to pay; disclosing the current amount may yet be another factor that affects the results.

Our findings of limited support for ODA for social development amongst the Hong Kong public is in contrast to similar surveys in other countries, and could relate to Hong Kong's status as a Special Administrative Region of China with no independent foreign policy. However, most respondents supported the concept of the DRF and supported allocating more resources to it. If this is true, this could imply that aid agencies and other humanitarian relief agencies could potentially have access to larger grants during natural disasters. It is possible that the definition of "disasters" could be expanded to cover shorter term humanitarian crises that are less visible than earthquakes or hurricanes e.g. acute food insufficiency during drought, or outbreaks of disease such as the recent outbreak of cholera in Yemen. Future surveys could indicate the current level of foreign aid to provide a clear picture of the existing relief situation, and separate the public support for foreign aid and public satisfaction with levels of foreign aid.

## Supporting information

S1 Appendix (DOC)

S2 Appendix (XLS)
