# Supplementary material for: Hong Kong’s role in global health: Public opinion of official development assistance
Source: PLoS One. 2018 Dec 4;13(12):e0207687. doi: 10.1371/journal.pone.0207687 (PMC6279225; doi:10.1371/journal.pone.0207687)
Supplement: S1 Appendix — (DOC) [file pone.0207687.s001.doc]

Hong Kong’s role in global health: public opinion of official development assistance

|  |  |  |  |  |  |
| --- | --- | --- | --- | --- | --- |
|  |  |  |  |  |  |
|  |  |  |  |  |  |
|  |  |  |  |  |  |
|  |  |  |  |  |  |
|  |  |  |  |  |  |
|  |  |  |  |  |  |
|  |  |  |  |  |  |
|  |  |  |  |  |  |
|  |  |  |  |  |  |
|  |  |  |  |  |  |
|  |  |  |  |  |  |
|  |  |  |  |  |  |
|  |  |  |  |  |  |
|  |  |  |  |  |  |
|  |  |  |  |  |  |
|  |  |  |  |  |  |
|  |  |  |  |  |  |
|  |  |  |  |  |  |
|  |  |  |  |  |  |
|  |  |  |  |  |  |
|  |  |  |  |  |  |
|  |  |  |  |  |  |
|  |  |  |  |  |  |
|  |  |  |  |  |  |
|  |  |  |  |  |  |
|  |  |  |  |  |  |

|  |  |  |
| --- | --- | --- |
|  |  |  |
|  |  |  |
|  |  |  |
|  |  |  |
|  |  |  |
|  |  |  |
|  |  |  |
|  |  |  |
|  |  |  |
|  |  |  |
|  |  |  |
|  |  |  |
|  |  |  |
|  |  |  |
|  |  |  |
|  |  |  |
|  |  |  |
|  |  |  |
|  |  |  |
|  |  |  |
|  |  |  |
|  |  |  |
|  |  |  |
|  |  |  |

|  |  |  |  |  |  |
| --- | --- | --- | --- | --- | --- |
|  |  |  |  |  |  |
|  |  |  |  |  |  |
|  |  |  |  |  |  |
|  |  |  |  |  |  |
|  |  |  |  |  |  |
|  |  |  |  |  |  |
|  |  |  |  |  |  |
|  |  |  |  |  |  |
|  |  |  |  |  |  |
|  |  |  |  |  |  |
|  |  |  |  |  |  |
|  |  |  |  |  |  |
|  |  |  |  |  |  |
|  |  |  |  |  |  |
|  |  |  |  |  |  |
|  |  |  |  |  |  |
|  |  |  |  |  |  |
|  |  |  |  |  |  |
|  |  |  |  |  |  |
|  |  |  |  |  |  |
|  |  |  |  |  |  |
|  |  |  |  |  |  |
|  |  |  |  |  |  |
|  |  |  |  |  |  |
|  |  |  |  |  |  |
|  |  |  |  |  |  |

|  |  |  |
| --- | --- | --- |
|  |  |  |
|  |  |  |
|  |  |  |
|  |  |  |
|  |  |  |
|  |  |  |
|  |  |  |
|  |  |  |
|  |  |  |
|  |  |  |
|  |  |  |
|  |  |  |
|  |  |  |
|  |  |  |
|  |  |  |
|  |  |  |
|  |  |  |
|  |  |  |
|  |  |  |
|  |  |  |
|  |  |  |
|  |  |  |
|  |  |  |
|  |  |  |

Appendix 1

**Survey questionnaire**

Interviewer to read out following statements: Hong Kong government allocates 18%, 18% and 14% of its budget to infrastructure, education and health respectively. The government's Disaster Relief Fund helps people in emergencies, e.g. earthquakes in Sichuan and Nepal while it currently gives no foreign aid to support long term social development (such as education and health) in low-income countries.

訪問員先讀出句子：香港政府既財政預算中有百分之18用喺基建，而百分之18同14就分別用喺教育同衛生方面。政府亦會動用救災基金去幫助受緊急情況影響既災民，例如四川同尼泊爾地震。另外，政府目前並無提供撥款去協助海外低收入國家喺教育和衛生等方面既長期社區發展。

**ODA-Q1**

Do you agree or disagree that some government budget should be allocated to the **Disaster Relief Fund**? If yes, what do you think would be the ideal percentage?

你同唔同意政府既財政預算應該撥款俾**救災基金**？如果同意，咁有關撥款應該佔財政預算既百分之幾？

| Agree, _________ % (two decimal places allowed, i.e. within 0.01-100.00)  Agree, but don’t know the ideal percentage  Disagree  Don’t know/hard to say/depends on the amount  Refuse to answer | 同意，佔百分之____________ (可答兩個小數位，即0·01-100·00之間)  同意，不知道百分比  唔同意  唔知／難講／視乎金額  拒答 |
| --- | --- |

**ODA-Q2**

Do you agree or disagree that some government budget should be allocated to the **Foreign Aid for Social Development**? If yes, what do you think would be the ideal percentage?

**你又同唔同意政府既財政預算應該撥款去支援海外低收入國家既長期社區發展？如果同意，咁有關撥款應該佔財政預算既百分之幾？**

| Agree, _________ % (two decimal places allowed, i.e. within 0.01-100.00)  Agree, but don’t know the ideal percentage  Disagree  Don’t know/hard to say/depends on the amount  Refuse to answer | 同意，佔百分之____________ (可答兩個小數位，即0·01-100.00之間)  同意，不知道百分比  唔同意  唔知／難講／視乎金額  拒答 |
| --- | --- |

**Demographics**

**DM1 Gender 性別**

Male 男
Female 女

**DM2 Age 年齡**

18-19

20-29

30-39

40-49

50-59

60-69

70 or above 70或以上

Refuse to answer拒絕回答

**DM3 Education level 教育程度**

Primary or below小學或以下

Secondary 中學

Tertiary: non-degree專上非學位

Tertiary: degree or above專上學位或以上

Refuse to answer拒絕回答

**DM4 Occupation職位**

Managers and administrators老闆/經理/行政人員

Professionals專業人員

Associate professionals輔助專業人員

Clerks文員

Service workers服務工作人員

Shop sales workers商店銷售人員

Skilled agricultural and fishery workers漁農業熟練工人

Craft and related workers手工藝及有關人員

Plant and machine operators and assemblers機台及機器操作員及裝配員

Drivers司機

Non-skilled workers非技術工人

Domestic helpers家庭傭工

Unclassified不能辨別

Others其他

Refuse to answer拒絕回答
